# Daily and seasonal fluctuation in Tawny Owl vocalization timing

**DOI:** 10.1371/journal.pone.0231591

**Published:** 2020-04-15

**Authors:** Patricia V. Agostino, Nicholas A. Lusk, Warren H. Meck, Diego A. Golombek, Guy Peryer

**Affiliations:** 1 Department of Science and Technology, National University of Quilmes/CONICET, Buenos Aires, Argentina; 2 Department of Psychology and Neuroscience, Duke University, Durham, NC, United States of America; 3 School of Health Sciences, University of East Anglia, Norwich, England, United Kingdom; University of Sassari, ITALY

## Abstract

A robust adaptation to environmental changes is vital for survival. Almost all living organisms have a circadian timing system that allows adjusting their physiology to cyclic variations in the surrounding environment. Among vertebrates, many birds are also seasonal species, adapting their physiology to annual changes in photoperiod (amplitude, length and duration). Tawny Owls (*Strix aluco*) are nocturnal birds of prey that use vocalization as their principal mechanism of communication. Diurnal and seasonal changes in vocalization have been described for several vocal species, including songbirds. Comparable studies are lacking for owls. In the present work, we show that male Tawny Owls present a periodic vocalization pattern in the seconds-to-minutes range that is subject to both daily (early vs. late night) and seasonal (spring vs. summer) rhythmicity. These novel theory-generating findings appear to extend the role of the circadian system in regulating temporal events in the seconds-to-minutes range to other species.

## Introduction

Vocalization is a complex behavior and crucial in language evolution [1]. Birds are the most vocal group of animals other than humans and a few other primates. In songbirds (order Passeriformes, suborder oscines), neural vocal control is achieved by a chain of interconnected brain areas in the fore‐, mid‐, and hindbrain [2]. Vocal communication is also of vital importance to non-oscine birds (owls, gulls, doves, etc.), which share with oscines a system of vocal control nuclei. In particular, owls (order Strigiformes) rely on acoustic signals for long-distance communication and hence are very vocal in different contexts [3].

In songbirds (e.g., zebra finches, canaries), the motor pathway for song proceeds from the high vocal center (HVC) to the nucleus robustus arcopallialis (RA), which then projects directly to vocal motoneurons (XIIts) and to respiratory premotor neurons in the nucleus retroambigualis (RAm) in the lower medulla. In turn, the RAm projects upon spinal motoneurons that innervate expiratory muscles (which provide the pressure head for vocalization), and upon XIIts to effect vocal–respiratory coordination [4,5]. In non-oscine birds, the syrinx has fewer muscles than that of oscines and in many cases intrinsic muscles are entirely absent. The non-oscine vocal control pathway has been scarcely investigated [6].

Owl vocalizations are simple compared with passerine vocalizations. An adult male canary, for example, has a repertoire of about 2 or 3 dozen different syllable types [7]. On the other hand, the predominant calls of the owls consist of simple notes or syllables that are highly stereotyped [8]. The complex vocal behavior exhibited by oscine songbirds is learnt by imitation of those from older members of their own species. Vocal learning is characterized by its dependence on intact hearing and a specialized forebrain circuitry that innervates vocal and respiratory nuclei of the brainstem. These behavioral and neuroanatomical attributes have not been found in non-vocal learners, which develop species-specific vocalizations in the absence of hearing, and have no known forebrain vocal motor control. In non-vocal learners, the vocal pathway is thought to consist solely of midbrain and brainstem nuclei [9].

Besides vocalizations, temporal information is also essential for communication. Biological time mechanisms comprise distinctive processes that span several orders of magnitude, from microseconds to seasonal events [10–12]. Among these temporal orders, almost all living organisms are subjected to the influence of the Earth’s rotational 24-h cycle. This rhythmic pattern, with a period close to 24-h, is called circadian rhythm (from the Latin words *circa dies*, around a day). The spectrum of rhythmic events is also subdivided into ultradian rhythms (with periods shorter than 24-h) and infradian rhythms (with periods longer than 24-h). In the seconds-to-minutes range, temporal discrimination, known as interval timing, is critical for fundamental behaviors such as foraging, decision-making and learning [13,14]. In addition, annual/seasonal cycles are fundamental for reproduction, migration and several physiological regulations in many species [15]. Besides the regulation of daily rhythms, the circadian clock is involved in photoperiodic time measurement.

Biological timing is essential in birds. Among vertebrate species, birds have highly sophisticated photoperiodic mechanisms that detect changes in daylength to adapt to seasonal environmental variations [16]. In the circadian range, robust nocturnal elevation in vocal activity has been previously documented in several bird species [17–19]. Most of the research related to circadian rhythmicity in vocalization has been performed in songbirds [19, 20]. However, both circadian and ultradian rhythmicity has previously been described in owls. For example, studies in the barn owl (*Tyto alba*) showed that nestlings depend on the daily regulation of stress hormones [21]. Moreover, ultradian rhythms in sleep-wakefulness were found in barn owlets [22].

The Tawny Owl (*Strix aluco*) is a crepuscular predator—with little activity during the day—that attacks vertebrate prey from perches in trees [23]. Tawny Owls are extensively distributed throughout the Eurasian continent, from Britain in Western Europe and northwest Africa to East and South Asia [24]. This work investigates whether the vocalization pattern of male Tawny Owls (*Strix aluco*), from hundreds of milliseconds to seconds range, is subjected to daily (early vs. late night) and seasonal (spring vs. summer) rhythmicity. Our study addresses the general hypothesis that, similar to songbirds, owl’s vocalization pattern may also present daily and seasonal variations. We present exploratory evidence indicating that phase regularity (i.e., Interval timing consistency between calls) presents both daily and seasonal variations.

## Materials and methods

### Experimental design

Territorial calls (also referred as hooting) of 30 male Tawny Owls (Strix aluco) in their natural environment were recorded in several European countries along different seasons and recording times (see Table 1). Most recordings were obtained from www.xeno-canto.org (Xeno-canto, XC), a non-profit website set up to share recordings of bird sounds worldwide [25], which has already been used for research purposes [26,27]. Only high quality recordings were chosen. Personal recordings were obtained by the corresponding author. Each recording contained calls of one focal owl, which serve both in territorial defense and mate choice. The recordings were made during comparable, favorable meteorological conditions (without strong winds or precipitation). The present study was purely observational and non-invasive, therefore no special permits were required.

**Table 1:** Mean values for *Strix aluco* vocalization patterns analyzed in the present study.

Owl	Source Information	Sound Recordist*	Country	Month	Time	Call1	Call2	T1	T2
						(s)	(s)	(s)	(s)
1	https://www.xeno-canto.org/358345	Xeira, A.	Germany	Feb	19:30	1.041	1.262	5.063	18.154
2	Personal recording	Peryer, G.	England	May	0:00	1.016	1.572	3.453	27.591
3	https://www.xeno-canto.org/302231	M, D.	England	March	5:24	0.810	1.149	6.287	20.809
4	Personal recording	Peryer, G.	England	May	0:00	1.004	1.258	5.768	17.484
5	https://www.xeno-canto.org/374099	Rossi, C.	Spain	May	-	0.919	1.209	6.958	15.808
6	https://www.xeno-canto.org/171690	Knychata, A.	Poland	March	22:30	1.027	1.309	5.985	20.941
7	https://www.xeno-canto.org/333455	Buhl, J.	Germany	June	5:00	1.142	1.404	5.552	23.063
8	https://www.xeno-canto.org/342461	Buhl, J.	Germany	June	4:00	1.368	1.622	5.516	39.291
9	https://www.xeno-canto.org/176220	Szczypinki, P.	Poland	April	22:00	0.860	1.070	6.084	12.670
10	https://www.xeno-canto.org/310463	Knychata, A.	Poland	April	22:30	1.095	1.244	5.731	15.014
11	https://www.xeno-canto.org/323831	Buhl, J.	Germany	June	4:00	1.437	1.587	5.573	22.986
12	https://www.xeno-canto.org/323832	Buhl, J.	Germany	June	4:30	1.228	1.420	5.380	23.168
13	https://www.xeno-canto.org/329497	Buhl, J.	Germany	June	21:30	1.389	1.642	5.441	21.591
14	https://www.xeno-canto.org/324439	Buhl, J.	Germany	June	4:00	1.147	1.333	5.368	27.426
15	https://www.xeno-canto.org/324428	Buhl, J.	Germany	June	4:00	1.163	1.326	5.289	23.797
16	https://www.xeno-canto.org/333452	Buhl, J.	Germany	June	4:00	1.338	1.574	5.689	26.668
17	https://www.xeno-canto.org/310638	Aberg, P.	Sweden	March	21:00	0.926	1.039	5.925	14.408
18	https://www.xeno-canto.org/310628	Aberg, P.	Sweden	March	19:00	0.866	1.207	6.989	17.247
19	https://www.xeno-canto.org/240986	Yablonovska-Grishchenko. E.	Ukraine	April	5:00	0.872	1.088	6.436	26.026
20	https://www.xeno-canto.org/208101	Melichar, D.	Czech Rep.	Nov	19:00	0.887	1.267	4.842	22.020
21	https://www.xeno-canto.org/115886	Ryberg, E.A.	Norway	Dec	17:00	0.998	1.247	5.895	16.034
22	https://www.xeno-canto.org/54025	Dragonetti, M.	Italy	Sept	22:00	0.814	1.110	5.166	14.968
23	https://www.xeno-canto.org/393976	Livon	Estonia	May	21:00	0.868	1.035	4.946	16.278
24	https://www.xeno-canto.org/346106	Buhl, J.	Germany	June	4:00	1.467	1.743	5.682	28.239
25	https://www.xeno-canto.org/298881	Brookes, C.	England	Jan	17:30	0.855	1.187	7.525	22.295
26	https://www.xeno-canto.org/293574	van Bruggen, J.	France	July	23:00	0.831	1.587	4.183	15.678
27	https://www.xeno-canto.org/198251	Matacz, L.	Poland	April	21:20	1.030	1.732	5.640	15.839
28	https://www.xeno-canto.org/165382	Tumiel, T.	Poland	April	0:30	1.173	1.443	4.656	19.745
29	https://www.xeno-canto.org/143531	Szczypinki, P.	Poland	April	21:00	1.600	1.754	4.555	12.319
30	https://www.xeno-canto.org/328946	Buhl, J.	Germany	June	4:00	1.221	1.263	5.371	31.524

* Sound recordists cited in accordance with Xeno-Canto terms of use under a Creative Commons license (www.xeno-canto.org/about/terms)

### Statistical analysis

**Fig 1:**
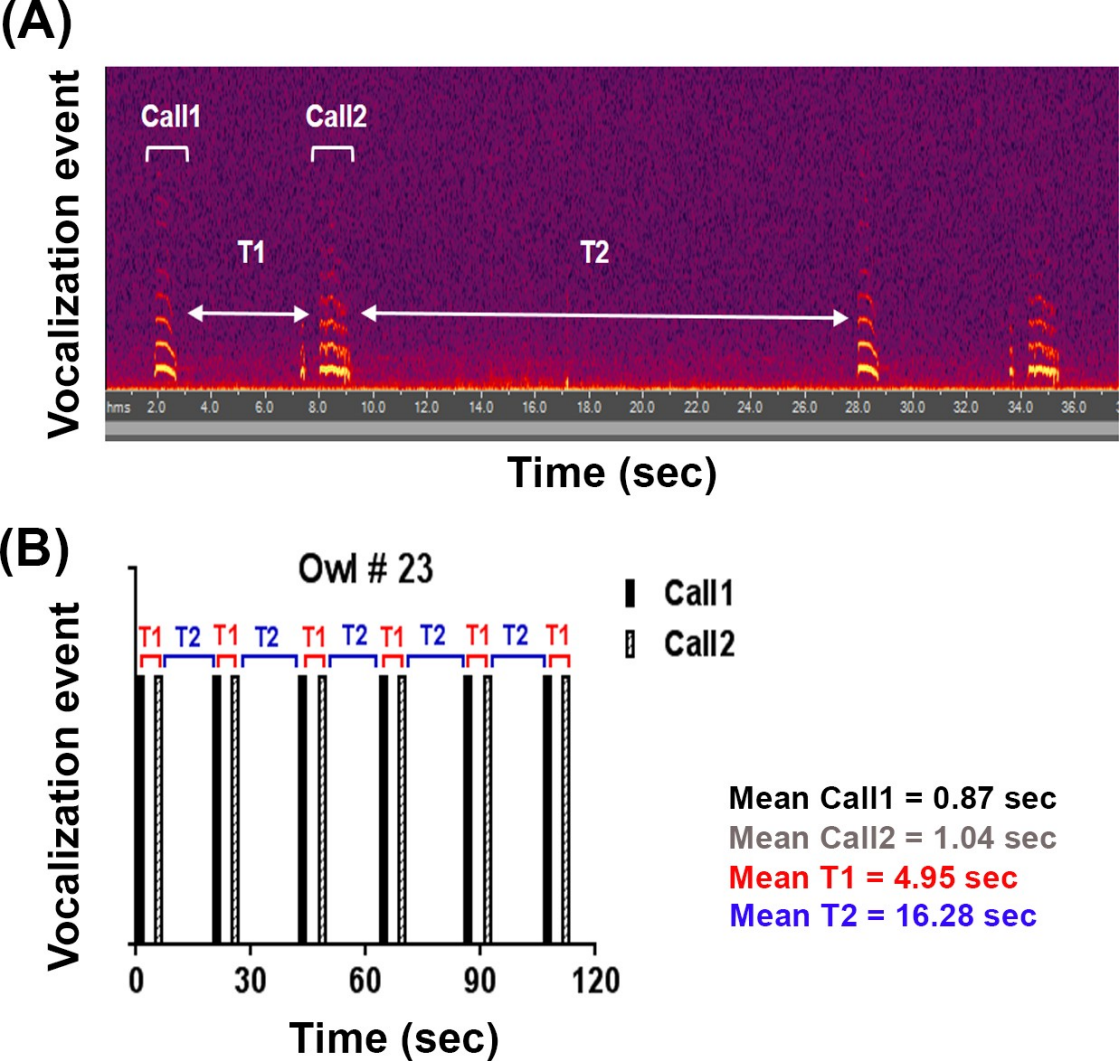
Vocalization pattern in male Tawny Owls. (A) Sonogram depicting the vocalization events of a single owl along time. Call1, Call2, T1 and T2 are shown. The ~ 200 msec vocal bout that precedes the second call (bottom arrow) was not present in all animals, and was not included in Call2. (B). Schematic representation of temporal vocalization events in a single owl, showing this pattern of two vocalizations (Call1-Call2) that is repeated as a function of time. The time interval between these two vocalizations is called T1, and the time interval between each repetition event is called T2. Mean values for each parameter were calculated per owl.

Digital sonogram analysis from audio files was performed by using Adobe Audition software (San Jose, California, USA). Male Tawny owls presented a distinctive pattern of two vocalizations (Call1-Call2) that was repeated over time (Fig 1). The time interval between these two vocalizations was defined as T1, and the time interval between each repetition event was named T2, as shown in Fig 1A and 1B. Mean times for Call1, Call2, T1 and T2 were calculated for each owl by taking the average times of recorded calls, with a minimum of 6 calls per owl (Fig 1B). We analyzed temporal variation (daily and seasonal) in these parameters. Tawny Owls have nocturnal or crepuscular habits and, in order to assess daily variations in vocalization, owls were divided into two groups: early owls (calls emitted between 17:00 and 0:00 h, n = 17) and late owls (calls emitted between 0:00 and 6:00 h, n = 12). Owl #5 was excluded from daily analysis because recording time is unknown. For seasonal analysis, owls were also divided into two groups: spring owls (recorded between February and May, n = 15) and summer owls (recorded between June and September, n = 12). Owls #20, #21 and #25 were excluded from seasonal analysis because they were not recorded in the above-mentioned months.

While the two vocalizations often contain distinct temporal and spectral profiles, the spectral differences were absent in Owls #6, #25, #31. Therefore, an unbiased classification of calls was done using an unsupervised DBSCAN clustering method utilizing Python’s sklearn package. The choice of this method over other classifiers was twofold. First, it does not require the number of clusters to be specified *a priori*. In addition to intervals T1 and T2, breaks in the typical vocal pattern may lead to other groupings based on temporal properties. This allows the detection of subtler complexities in vocalization structure. Second, it can detect arbitrarily shaped clusters. As the variance of the model parameters may not be equal, this allows the model to be run with minimal preprocessing or distortion of vocalization data. Classification was done using all calls with two model features related to the temporal components of the vocalization: duration of call (in seconds) and time between calls (also in seconds). All call data was used allowing classifications to be validated against calls with available spectral data. The model parameters for maximum distance between samples (Eps) and the minimum number of samples in a neighborhood (MinPts) were set to 0.9053 and 7, respectively, and the distance metric was Euclidean. Parameterization was done in accordance with previous research [28]. In short, MinPts was first set using the heuristic MinPts ≈ ln(n), rounded to the nearest whole number, where n is the number of samples. Eps is then calculated by first finding the distances between *k-nearest neighbors*, with *k* set to MinPts. These values are then sorted in descending order and the point of maximum curvature is assigned to Eps.

Daily (early vs. late night) and seasonal (spring vs. summer) data were evaluated using a two-tailed t-test. When equality of variances was not met, Welch’s corrections were applied.

Statistical analyses were performed using Graphpad Prism (GraphPad Software Inc., CA, USA). In all cases, the alpha level was set at 0.05.

## Results

Our findings indicate that the temporal structure of Tawny Owl vocalizations—in the seconds-to-minutes range of interval timing—presents both daily and seasonal variation.

Table 1 displays the mean times for the parameters Call1, Call2, T1 and T2 (Fig 1 and Methods) of all 30 owls evaluated. In total, Call2 was longer than Call1, presenting a mean time of 1.36 ± 0.22 sec (mean ± S.D.), while mean time for Call1 was 1.08 ± 0.22 sec. (t_29_ = 9.768, p<0.0001, two-tailed paired t-test). Interval times T1 and T2 presented an average of 5.57 ± 0.82 sec and 20.95 ± 6.12 sec, respectively (t_29_ = 13.51, p<0.0001, two-tailed paired t-test). Moreover, T2 intervals presented higher variability compared to T1. In this sense, the coefficient of variation (CV) for the 30 owls evaluated was higher for T2 than T1 (t_29_ = 7.211, p<0.0001, two-tailed paired t-test, S1 Fig). An analysis of the geographical distribution of all data evaluated indicated no significant effect of region in all four parameters analyzed (S2 Fig).

As not all owls emit a short vocalization prior to Call2 (Fig 1A) and occasional breaks in the vocalization pattern lead to repeated call types, an unsupervised clustering algorithm was run on interval times. This allowed for unbiased classification of intervals that may be distinct from T1 and T2 distributions (S3 Fig). The data were found to have four discrete clusters: initial calls, T1 calls, T2 calls and long T2 calls.

As short prior vocalizations are unique to Call2, the accuracy of the classifier was assessed by comparing the number of intervals identified as T1 against known T1 intervals that were followed by an identifiable Call2. The classifier showed exceptional accuracy by correctly identifying all 286 T1 intervals in owls that emitted short vocalizations prior to Call2 and only 9 of 325 as T1 intervals when it was not followed by a Call2, giving a classification accuracy of 98.5%. Interestingly, despite the difference in spectral composition, there was no significant difference between the duration of T2 calls with or without short vocalizations prior (t_29_ = −0.404, p = 0.689, two-tailed unpaired t-test).

**Fig 2:**
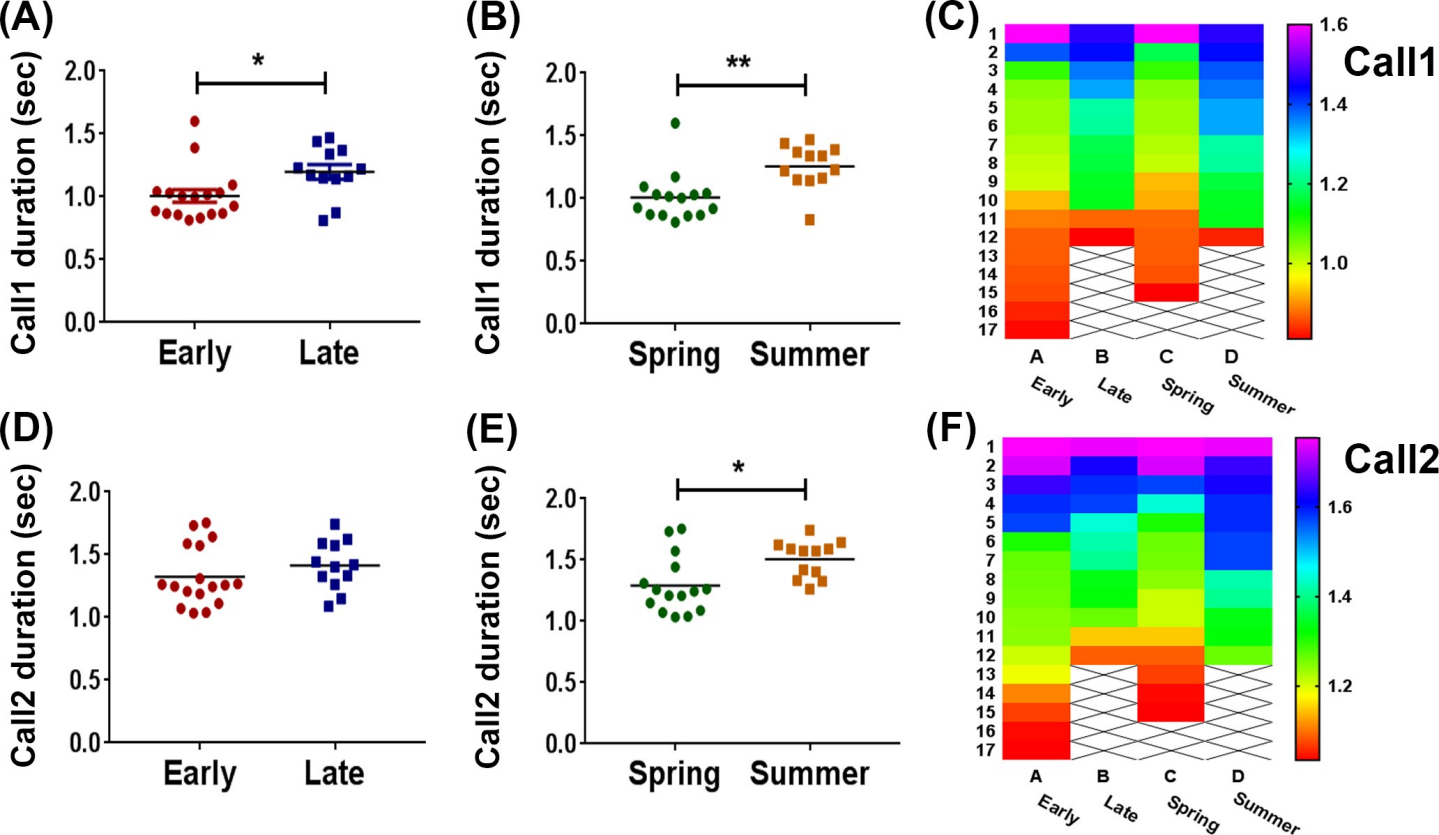
Daily and seasonal variation in call duration. (A) Call1 duration in early vs. late owls (p = 0.0197), (B) Call1 duration in spring vs. summer owls (p = 0.0019), (C) Heat map for Call1. (D) Call2 duration in early vs. late owls (p = 0.3043). (E) Call2 duration in spring vs. summer owls (p = 0.0109), (F) Heat map for Call2. Data from scatter dot plots represent the mean value for each owl. In heat maps, each row corresponds to the mean value per owl, and the columns represent the different groups (n = 17 for early owls, n = 12 for late owls, n = 15 for spring owls, and n = 12 for summer owls). **p<0.01, *p<0.05, two-tailed Student t-test.

Daily and seasonal analysis was performed for the parameters Call1, Call2, T1 and T2. For that purpose, owls were classified as early vs. late and spring vs. summer (see Methods). Call1 presented significant time of day and seasonal effects. As shown in Fig 2, Call1 duration was shorter in early vs. late owls (t_27_ = 2.481, p = 0.0196, two-tailed unpaired t-test, Fig 2A). A similar effect was observed in spring vs. summer owls for both Call1 (t_25_ = 3.464, p = 0.0019, two-tailed unpaired t-test, Fig 2B) and Call2 (t_25_ = 2.749, p = 0.0109, two-tailed unpaired t-test, Fig 2E). On the other hand, there were no time of day differences in Call2 duration (t_27_ = 1.047, p = 0.3043, two-tailed unpaired t-test, Fig 2D). Cluster heat maps were generated from these data to visually represent the increase or attenuation in call duration across the different groups. Fig 2C and 2F display heat maps containing the mean value per owl (colored square) for Call1 and Call2, respectively. Color rank for Call1 is clearly different for early vs. late owls, as well as for spring vs. summer owls, while Call2 color rank is visibly different only in spring vs. summer owls. Additionally, the coefficient of variation (CV) for Call1, calculated as the ratio between the standard deviation and the mean, was significantly increased in summer vs. spring owls (t_25_ = 3.063, p = 0.0052, two-tailed unpaired t-test).

**Fig 3:**
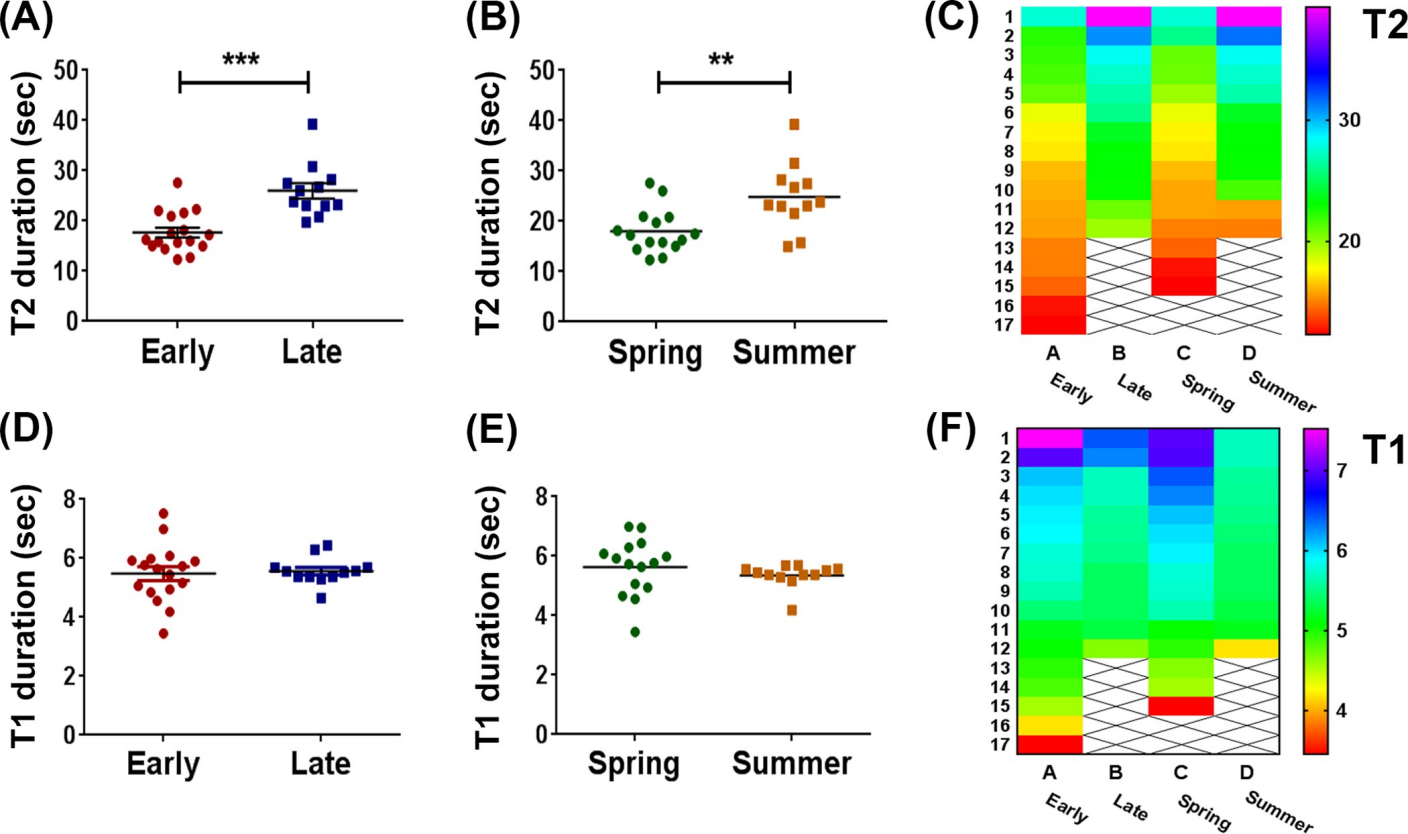
Daily and seasonal variation in timing between calls. (A) T2 duration in early vs. late owls (p<0.0001), (B) T2 duration in spring vs. summer owls (p = 0.0037), (C) Heat map for T2, (D) T1 duration in early vs. late owls (p = 0.7574), (E) T1 duration in spring vs. summer owls (p = 0.3147), (F) Heat map for T1. Data from scatter dot plots represent the mean value for each owl. In heat maps, each row corresponds to the mean value per owl, and the columns represent the different groups (n = 17 for early owls, n = 12 for late owls, n = 15 for spring owls, and n = 12 for summer owls). ***p<0.0001, **p<0.01, two-tailed Student t-test.

Owls also displayed significant time of day and seasonal effects in T2, as shown in Fig 3. Calls from late owls presented longer T2 intervals compared to calls from early owls (t_27_ = 4.849, p<0.0001, two-tailed unpaired t-test, Fig 3A). T2 also exhibited a seasonal variation, with longer values in summer vs. spring owls (t_25_ = 3.228, p = 0.0035, two-tailed unpaired t-test, Fig 3B). These differences in T2 can also be observed in the cluster heat map shown in Fig 3C. There were no effects of time of day or season in T1 values (t_24_ = 0.3124, p = 0.7574 for time of day, Fig 3D; t_19_ = 1.032, p = 0.3147 for season, Fig 3E, two-tailed unpaired t-test with Welch's correction; cluster heat map in Fig 3F). However, both standard deviation (SD) and coefficient of variation (CV) for T1 were significantly increased in early vs. late owls (t_24_ = 2.718, p = 0.0134 and t_24_ = 2.646, p = 0.0166, respectively, two-tailed unpaired t-test with Welch's correction).

**Fig 4:**
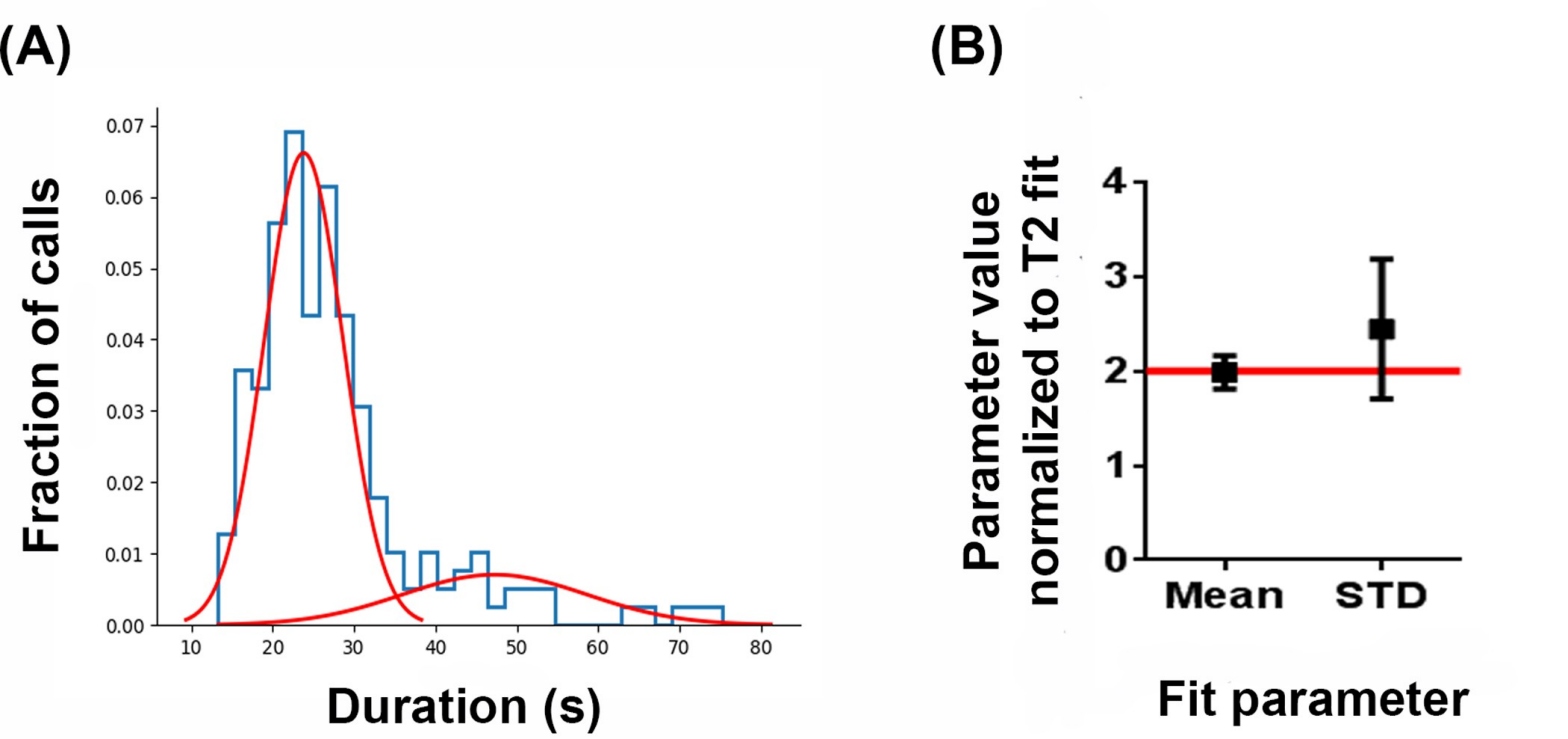
Distribution of T2 and Long T2 intervals. (A) Histogram and individual Gaussian fits for T2 and Long T2 intervals. (B) Scatter plot of the mean and 95% CI of the credible parameter space for the Long T2 intervals’ Gaussian Fit normalized to those of the T2 interval fit. Solid red line indicates the value that would be twice that of the T2 fit.

With the duration of T2 intervals falling into two distinct groups (i.e. T2 and long T2), the existence of a multiplicative relationship between the mean time and standard deviation of these populations was assessed. Due to the sparseness of long T2 calls, a Bayesian approach for estimating the population’s distribution was used. Both the mean and standard deviation of long T2 calls were found to not be significantly different than twice that of the T2 call distribution (Fig 4). Some examples of distribution of T2 and long T2 interval times around the median are shown in S4 Fig.

## Discussion

Vocal communication has an important biological function in male owls and is used for attracting females as well as establishing territory. Previous analysis of the frequency and temporal components of the calls made by male Tawny Owls has suggested a relationship with the health and fitness of the owl as indexed by parasite burden [29]. A number of factors are known to influence the vocal activity pattern of nocturnal birds. One factor is the time of the year, with calling rate varying along the breeding cycle because of the territorial/mating functions of calls [30–32]. Another factor is the time of day, with most owl species being more vocally active during dusk and dawn [17]. Thus, circadian changes in vocal production have been displayed in non-oscine birds such as the domestic Japanese quail (*Coturnix coturnix japonica*) [18]. Moreover, melatonin—the major timekeeping hormone in vertebrates—affects the temporal pattern of vocal signatures in both the oscine bird zebra finch (*Taeniopygia guttata*) and the non-oscine Japanese quail [19]. Melatonin is also relevant for vocal communication in the midshipman fish. Nocturnal fish vocalizations follow both daily and circadian rhythmicity under a light/dark cycle and constant dark conditions, respectively, and are rescued by melatonin under constant light [33]. These studies in birds and fish show daily and/or seasonal variations on comparable time scales as used in the present work. However, such investigations have not been previously researched in owls.

There are several indications that interval timing is present in songbirds but, again, there is no information on the subject in owls. Indeed, it has been proposed that the syllable can be used as a reliable time marker in order to predict song completion [34]. Calls in non-oscine birds are also temporally structured. In particular, male Tawny Owl calls present an organized structure of two clear vocalizations repeated over time, with a mean fundamental frequency below 1 kHz [35]. Call duration and silent intervals between these male Tawny Owl vocalizations fit in the seconds-to-minutes range of interval timing. Here we found a remarkable diurnal (early vs. late night) and seasonal (spring vs. summer) variation in parameters Call1 and T2, as well as seasonal variation in parameter Call2. Specifically, the time interval between each 2-call repetition event (T2) was around 18 sec in early owls and around 26 sec in late owls. These data indicate that time of day regulates the timing between calls. In a similar way, seasons influence the length of T2, being around 18 sec for spring owls and 25 sec. for summer owls. T1, on the other hand, remained mostly invariant around 5 sec.

This is the first description of such changes in non-oscine birds. In songbirds, syllables, intervals, motor control, and respiratory pathways have been well described [36]. Moreover, a clear circadian variation in song and calling behavior was found in zebra finches, controlled by pineal melatonin signaling [37]. Because we have previously demonstrated a role for melatonin in the circadian-interval timing interaction in other models [38], it is tempting to speculate that the pineal gland might influence the temporal allocation and short-length duration of owl vocalizations. Although this is beyond of the scope of the present study, the biological function of daily and seasonal variation in Tawny Owl vocalizations may be related to the breeding and territorial behaviors, ensuring the maximal survival of their offspring [16].

Some limitations of the present study must be considered. First, since only high-quality recordings were analyzed, our dataset is not widely distributed over the seasons (e.g., the summer season presents fewer recordings from July to September compared to June). Second, recordings were not performed under standardized or laboratory conditions. However, any fluctuations in recording distance or vegetation that may induce differences in reverberation times are relatively small for the fundamental frequencies recorded (close to 1 KHz) [39, 40], and therefore do not affect the parameters evaluated in the scale of interval timing. Finally, although we have no information related to the age of the animals evaluated, the parameters of Tawny Owl calls make individuals traceable over years [8], indicating vocal consistency within members of this species. Despite the listed methodological limitations, a clear variation in the temporal structure of calls can be identified.

Given that a robust relationship between the circadian oscillator and events in the seconds-to-minutes range has been previously established [41–44], these results contribute to the expansion of the role of the circadian system in regulating the shorter-duration temporal events mediated by the interaction of “time cells” in the cerebellum, striatum, and hippocampus [11, 45, 46]. Moreover, these novel findings take the lead in establishing a fundamental relation between interval timing in the seconds-to-minutes range and daily photoperiod as a function of the annual/seasonal cycle [47]. We provide a foundation for future studies of owl vocalizations under standardized conditions which may examine the observed temporal patterns in greater detail. Collaborating with contributors to existing databases of nature recordings may lead the way to more powerful field research to further understand temporal determinants of behavior.

## Supporting information

S1 Fig Coefficient of variation for T1 and T2. Coefficients of variations for T1 and T2 interval times in individual owls. t_29_ = 7.211, p<0.0001, two-tailed paired t-test, N = 30.
(TIF)

S2 Fig Geographical distribution of Tawny Owl recordings. (A) Map showing the European countries where Tawny Owl calls were recorded (see Table 1). Individual countries were pooled (as indicated by color) by proximity and number of recordings. No significant differences were observed for (B) Call1 (p = 0.0567), (C) Call2 (p = 0.0931), (D) T1 (p = 0.7245) and (E) T2 (p = 0.0564) Data are shown as mean ± S.D. One-way ANOVA.
(TIF)

S3 Fig Unsupervised clustering of between call intervals. (A) Classification of between call intervals using an unsupervised DBSCAN clustering method with length between calls (sec) and duration of calls (sec) as model features. (B) Comparison of intervals for calls that occurred in their proper sequence (blue) verses out of sequence (orange). No significant difference was found between these two groups for either T1 (p > 0.05), T2 (p > 0.05), or Long T2 (p > 0.05) intervals. Bonferroni corrected two-tailed Student t-test.
(TIF)

S4 Fig Distribution of T2 and long T2 interval times. Distribution of T2 times around their median in individual owls. Blue color data points represent values that exceed 360° (the median value); red color data points represent values that exceed 720° (more than twice the median value); and green color data points represent values that exceed 1080° (more than three times the median value).
(TIF)
