# Supplementary figures and images for: Daily and seasonal fluctuation in Tawny Owl vocalization timing
Source: PLoS One. 2020 Apr 15;15(4):e0231591. doi: 10.1371/journal.pone.0231591 (PMC7159226; doi:10.1371/journal.pone.0231591)

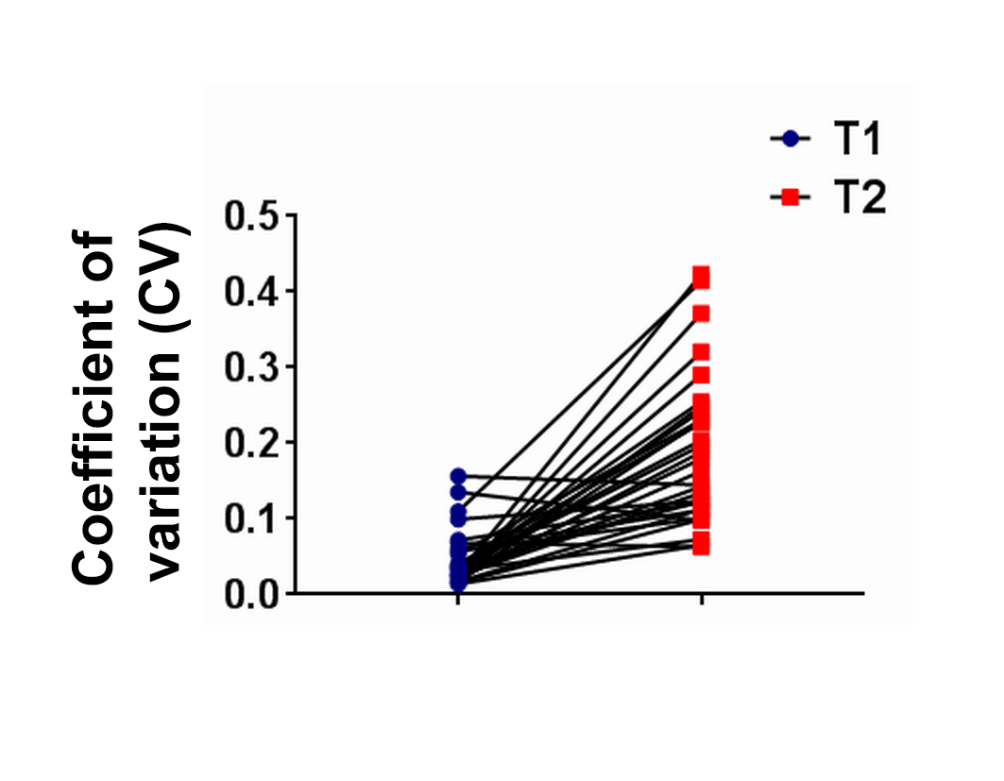

Supplement: S1 Fig — Coefficients of variations for T1 and T2 interval times in individual owls. t29 = 7.211, p<0.0001, two-tailed paired t-test, N = 30. (TIF) [file pone.0231591.s001.tif]

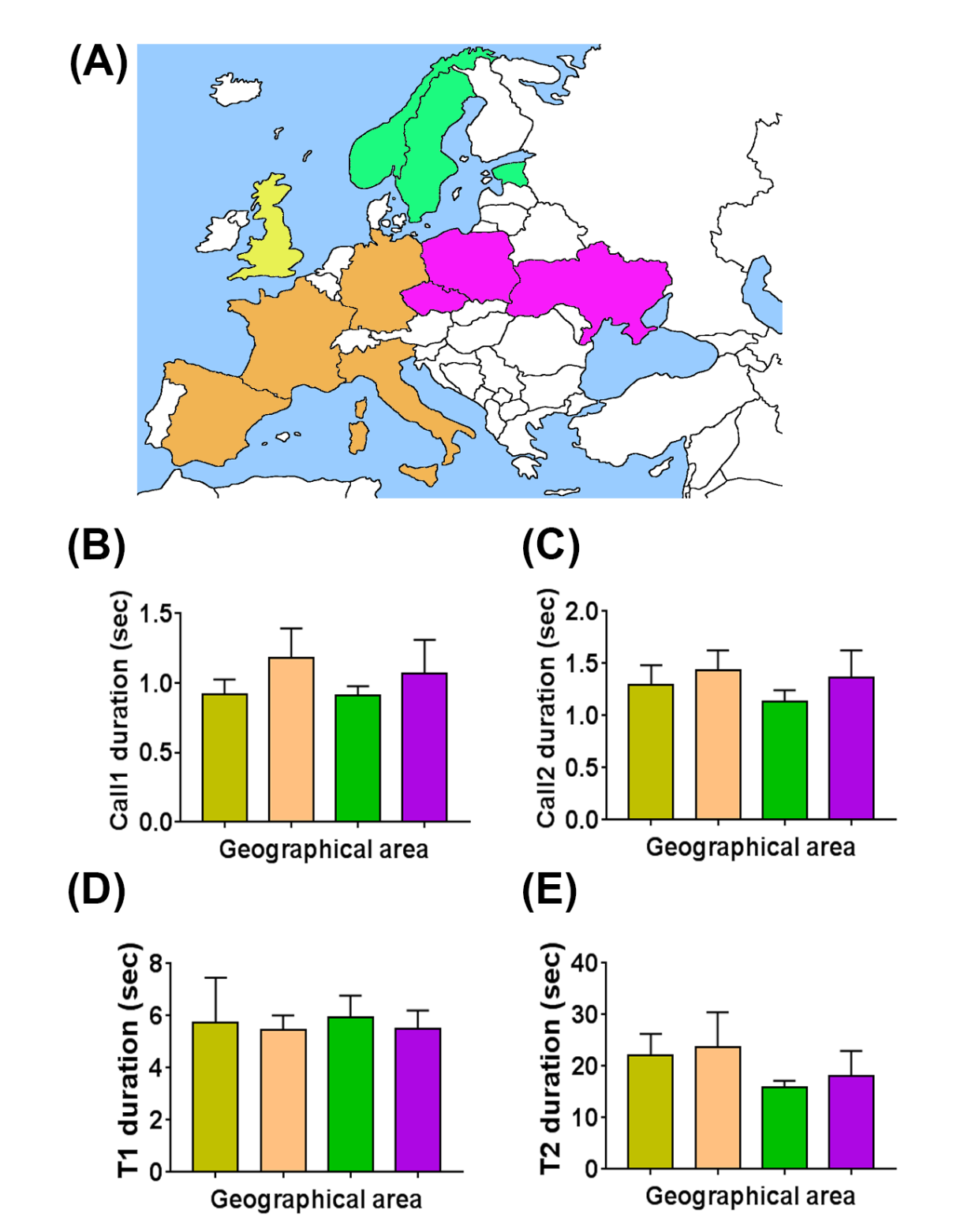

Supplement: S2 Fig — (A) Map showing the European countries where Tawny Owl calls were recorded (see Table 1). Individual countries were pooled (as indicated by color) by proximity and number of recordings. No significant differences were observed for (B) Call1 (p = 0.0567), (C) Call2 (p = 0.0931), (D) T1 (p = 0.7245) and (E) T2 (p = 0.0564) Data are shown as mean ± S.D. One-way ANOVA. (TIF) [file pone.0231591.s002.tif]

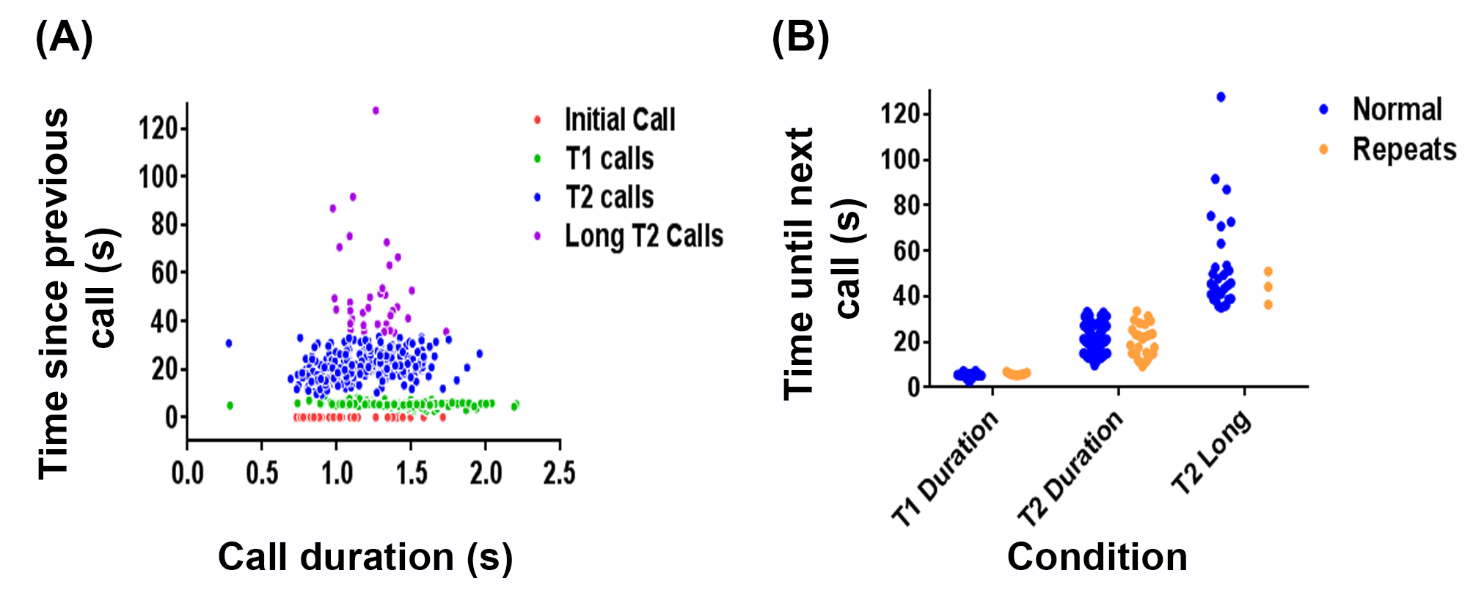

Supplement: S3 Fig — (A) Classification of between call intervals using an unsupervised DBSCAN clustering method with length between calls (sec) and duration of calls (sec) as model features. (B) Comparison of intervals for calls that occurred in their proper sequence (blue) verses out of sequence (orange). No significant difference was found between these two groups for either T1 (p > 0.05), T2 (p > 0.05), or Long T2 (p > 0.05) intervals. Bonferroni corrected two-tailed Student t-test. (TIF) [file pone.0231591.s003.tif]

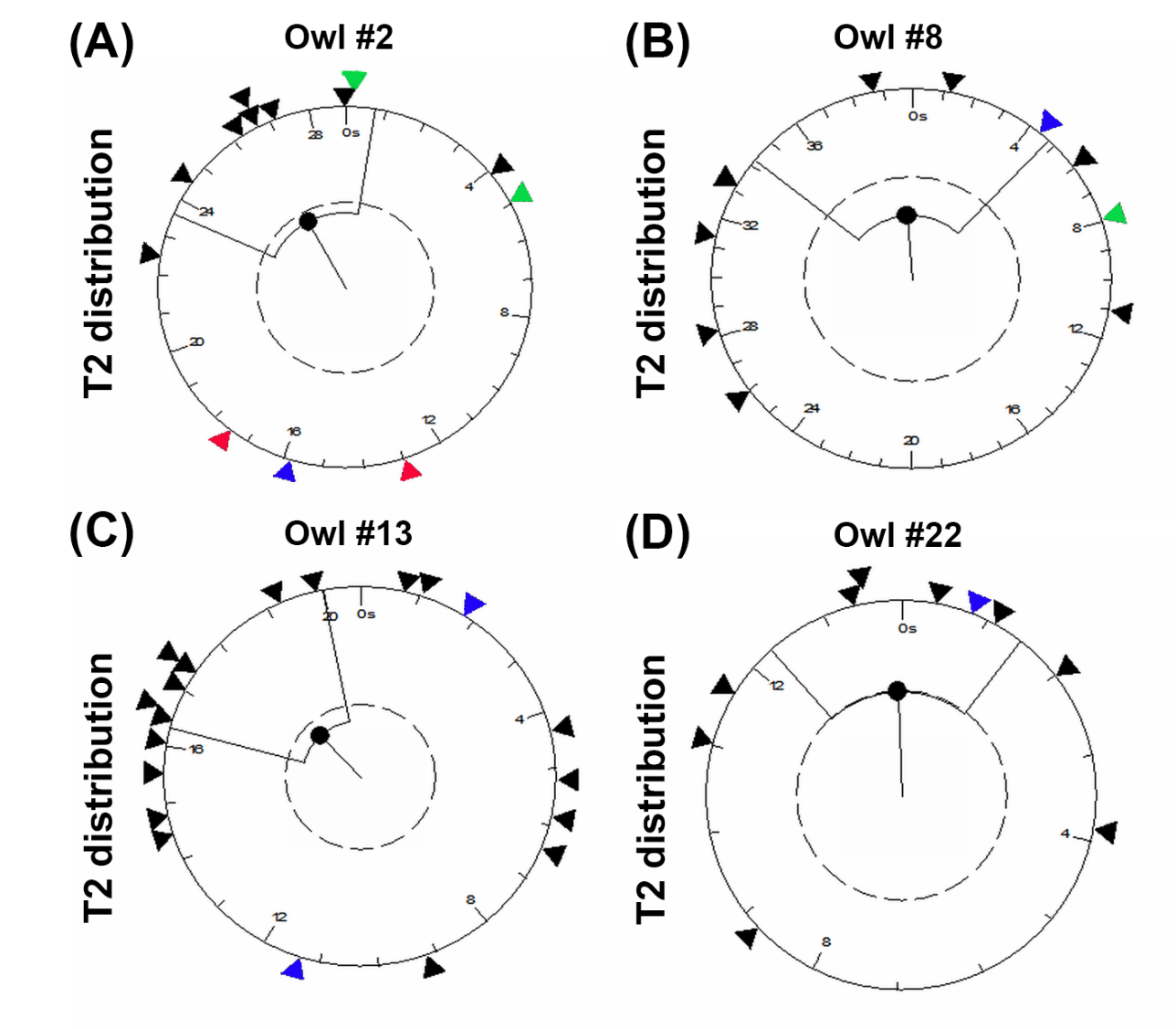

Supplement: S4 Fig — Distribution of T2 times around their median in individual owls. Blue color data points represent values that exceed 360° (the median value); red color data points represent values that exceed 720° (more than twice the median value); and green color data points represent values that exceed 1080° (more than three times the median value). (TIF) [file pone.0231591.s004.tif]
